# Field Traction Performance Test Analysis of Bionic Paddy Wheel and Vaned Wheel

**DOI:** 10.3390/biomimetics7040185

**Published:** 2022-11-03

**Authors:** Long Xue, Baofeng Xie, Fei Lin, Song Cheng, Lan Li, Muhua Liu, Jing Li

**Affiliations:** 1 Jiangxi Key Laboratory of Modern Agricultural Equipment, College of Engineering, Jiangxi Agricultural University, Nanchang 330045, China; 2 Key Laboratory of Optics-Electrics Application of Biomaterials of Jiangxi Province, Jiangxi Agricultural University, Nanchang 330045, China

**Keywords:** paddy field soil, bovine hoof surface, bionic walking-wheel, traction trafficability, slip coefficient

## Abstract

In order to improve the traction performance of a wheel of a micro-tiller on the soil surface of a paddy field, we extracted the surface curve of a cow’s hoof and used the cow’s hoof as a bionic prototype to design a bionic paddy wheel. In order to verify the passability of the bionic paddy wheel in paddy soil, a wheel-soil test bench was built in the experimental field with a moisture content of 36%. The test results show that under the condition of the same load on the wheel, the changing laws of torque, drawbar pull, and slip ratio of the bionic paddy wheel and the conventional vaned wheel are similar. The torque and drawbar pull of the bionic paddy wheel are higher than those of the conventional vaned wheel at the same slip ratio. The maximum torque and hook traction provided by the bionic paddy wheel and the conventional vaned wheel both increase with the increase of the load on the wheel. Under the same load on the wheel, the bionic paddy wheel is at least 22% higher than the conventional vaned wheel. Compared with the conventional vaned wheel, the bionic paddy wheel can provide a higher driving force and hook traction, which can improve the working efficiency of the vehicle in the paddy field.

## 1. Introduction

Jiangxi Province is an important commodity grain-producing area in China, known as ‘Jiangnan Granary’. From 2021, the province’s grain area will be 56.592, 6 million mu, the total output will be 21.923 billion kg, and the annual output will remain above 21.5 billion kg for nine consecutive years. Jiangxi Province is located in the south of the Yangtze River, with low latitude. The normal landform types are mainly mountains and hills. The landform can be summarized as: ‘six mountains and one water, two fields, one road and manor’, which belongs to the typical red and yellow soil low hills. However, due to the complex agricultural operation environment in hilly and mountainous areas, especially the cultivated land for grain crops such as terraced rice, because the terraced rice field is small (about 200 ^m2^), the gap is large, and it is narrow and long. Therefore, the design of light and simple, advanced, and applicable equipment to meet the hilly terraced rice operation has become the first choice to solve this problem. Micro tillage machines have the advantages of light weight, flexible operation, strong adaptability and high-cost performance. They are the main power machinery used for small plot tillage. When combined with a rotary tiller, direct seeding machine, ditching machine and other agricultural machinery, they can realize the tillage of terraced rice and the orderly planting of seedlings. When the wheels of conventional micro-cultivators drive and operate on the surface of paddy fields, due to the slip and serious subsidence of the wheels, the adhesion is small and the driving resistance is large. There are problems of poor trafficability, low efficiency, high energy consumption and inability to drive. Therefore, the design of a micro-cultivator wheel suitable for paddy field walking is conducive to improving the working efficiency of the micro-cultivator and reducing wheel slip and driving resistance.

In recent years, in order to improve the trafficability of vehicles or robots on the soft soil surface, domestic and foreign scholars have explored and studied the traction performance and subsidence mechanism of wheel foot forms of different types of wheel legs, walking wheels and convertible walking wheels. Goldman et al. from the Georgia Institute of Technology designed a six-wheeled robot, Sandbots, based on how lizards move across the desert to improve the ability to pass through soft sand [1]. Francisco et al. from the University of Surrey designed a walking wheel composed of five evenly distributed wheel legs. Each wheel leg is equipped with different types of foot-end structures to increase the contact area with the ground and has a strong passing ability [2]. The Whegs series of robots developed by Kathryn A. Daltorio et al. from Case Western Reserve University in the United States split the wheel into three-spoke walking wheels, with cylindrical feet in contact with the ground to overcome obstacles through changes in its flexible body [3]. Asguard is a five-spoke walking wheel robot developed by the German Artificial Intelligence Research Center, with semi-cylindrical rubber feet for climbing stairs [4]. Messor II, a wheel-legged robot developed by Dominik Belter et al. at Poznan University of Technology, has a hemispherical foot-end structure. The robot can cross obstacles by establishing a foot–soil perception model [5]. ANYmal quadruped robot was designed by Kolvenbach of ETH Zurich; in order to improve its stability, the foot structure is rectangular, and the robot can realize the inspection of underground pipe networks [6]. Chen took the lead in carrying out the research on ground mechanics of legged robots, and put forward the theory of bionic paddy wheels such as ‘half walking wheel‘, ‘convertible walking wheel‘ and ‘mechanical transmission walking wheel‘ [7]. Professor Zhuang et al. analyzed the mechanism of camels crossing sand in detail, and designed the imitation camel hoof tire to reduce the grounding specific pressure, restrain the sand flow under the wheel, and improve the propulsion and bearing capacity. Professor Gao Feng from Beijing University of Aeronautics and Astronautics proposed the concept of a deformable wheel on the basis of summarizing a variety of walking wheel movements. It can be converted between two wheel states with and without a rim to achieve walking and continuous walking [8]. Ding et al. [9] established a foot–ground interaction mechanical model based on the theory of ground mechanics, and verified the proposed model by experiments.

As a typical soft ground, paddy soil has large viscosity and relatively complex internal interactions. Its mechanical properties are sensitive to water content and compactness. A slight change in water content will lead to a greater change in the mechanical properties of paddy soil. Natural selection leads to some species can adapt to different geographical environments. For example, camels and ostriches live in the desert [10,11]. They have a unique foot-shaped structure that allows them to walk freely in the desert. Cattle is a long-term animal used for paddy field operation. The foot of the cattle hoof is petal-shaped. When it enters the soil, it enters the soil vertically as a whole with little resistance. After entering the soil, the foot is separated, the area increases, and the grounding pressure is reduced [12,13,14].

Therefore, in this paper, the cow hoof was used as the bionic prototype. By analyzing the influencing factors and main parts of the soil fixation and anti-slip of the cow hoof, the reverse engineering software was used to extract the characteristic elements with high passing capacity and low resistance in the cow hoof and apply the bionic elements to the wheel design. A bionic paddy wheel suitable for soft ground such as a paddy field was obtained and compared with the traction performance of a conventional paddy wheel in paddy field soil to verify the traction performance of the bionic paddy wheel.

## 2. Materials and Methods

### 2.1. Preparation of Bionic Structure

**Figure 1:**
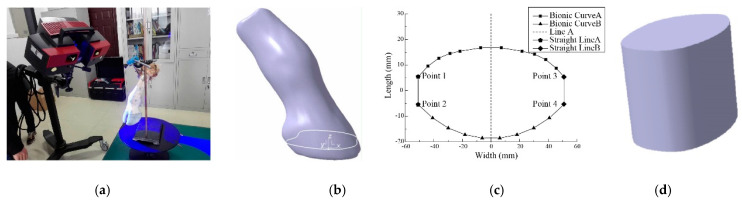
Design process of bionic structure. (**a**) data acquisition, (**b**) point cloud processing, (**c**) bionoc curve, (**d**) bionocal structure.

An adult healthy cattle hoof was selected as the research object, and the three-dimensional data point cloud of the whole cattle hoof surface was scanned by the three-dimensional optical scanner ATOS Triple Scan (precision 0.03 mm) (GOM Metrology, Braunschweig, Federal Republic of Germany). The point cloud was deleted, denoised, and filled with defects. After that, the operations of filling holes, removing features, grid doctor, simplification and relaxation were performed, and the three-dimensional features such as the hoof flap gap and hanging hoof were removed, as shown in Figure 1a. According to the simplified morphological characteristics of the hoof, it is mainly divided into two parts, namely the buried surface and the unearthed surface. The bottom surface of the hoof is regarded as a horizontal plane, and the three-dimensional coordinate system of the hoof was established with the maximum circumscribed circle center of the hoof bottom surface as the coordinate origin, as shown in Figure 1b. The characteristic curve segment at the maximum outer diameter of the lower end of the hoof was extracted, and the Bionic Curve A and Bionic Curve B curves were selected. After scaling the characteristic curve, the feature point 1 and the feature point 2 were connected by a straight line, and the feature point 3 and the feature point 4 were connected by a straight line. Then the coordinate origin was transformed to the center of the closed curve, and finally the coordinate points of the curve segment were exported to MATLAB. The derived characteristic curve was symmetrical in line A as shown in Figure 1c. The bionic paddy wheel foot model was established, as shown in Figure 1d.

The Bionic Curve A and Bionic Curve B in Figure 1c were curve-fitted, and the polynomial equation structure of the bionic curve is shown in Formula (1).

(1) $$F(x)=ax^{4}+bx^{3}+cx^{2}+dx+e$$

The coefficients of the two bionic curves are listed in Table 1. It can be seen from the table that the fitting correlation coefficients of the two bionic curves are 0.998 and 0.999, respectively.

**Figure 2:**
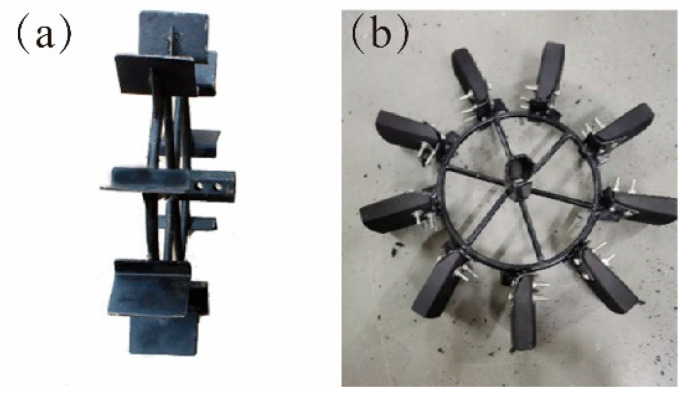
Test wheel. (**a**) Vaned wheel, (**b**) Bionic paddy wheel.

The raw material of the bionic structure is rubber blocks. The rubber block has the characteristics of convenient processing, strong compressive strength, not being easy to deform, and being lightweight and of low cost. With the wheel frame of the mini-cultivator as the carrier, eight L-shaped supporting plates were welded at equal intervals on the rim of the frame, mounting holes were machined on the supporting plates, and the bionic structure was fixed to the L-shaped supporting plate of the vaned wheel by bolts. Figure 2 shows the vaned wheel with blade device and the bionic paddy wheel with a bionic structure. The maximum diameter and wheel width of the two wheels in Figure 2a,b are equal, which are 740 mm and 102 mm respectively.

### 2.2. Test Rig Construction

**Figure 3:**
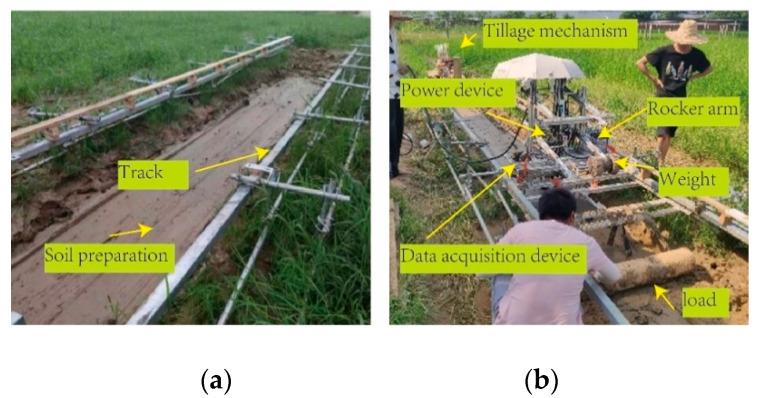
Test bench. (**a**) Track device, (**b**) Single wheel bench.

The test site was selected in the experimental field of Jiangxi Agricultural University. The steel pipe was used as the support frame of the track. The slide rail was fixed at both ends by hinges. The height and spacing of the two slide rails were adjusted. The total length and width of the track were 8.0 m and 1.2 m, respectively. Through the tillage device, the soil was first rotary tilled, and then scraped with a scraper. The soil after scraping is shown in Figure 3a. A single wheel bench was installed on a horizontal track, which mainly comprises a power device, a data acquisition device and a load-applying device (load). The power unit is mainly provided by a servo motor with a power of 1.5 kw, which transfers power to the wheels via a 1: 50 reducer. The data acquisition device mainly collects wheel torque, wheel horizontal moving actual speed and theoretical speed and wheel sinkage, and realizes system control and data acquisition through Arduino1.8; the load application device is a rocker’s arm structure. The load on the wheel is changed by changing the weight of the rear weight of the rocker arm, and the change of drawbar pull is realized by dragging the load of different weights.

The soil water content is 36.98 g (100 g), and its mechanical properties are shown in Table 2.

### 2.3. Experimental Design

The theoretical speed of the test wheel in this paper is 0.36 m/s. The data acquisition device collects the actual speed and theoretical speed of the wheel through the encoder. The on-wheel loads of the test wheels are 58.57 N, 84.57 N and 107.4 N respectively, and the change of the on-wheel load is controlled by adding or removing weights on the rocker’s arm. We changed the load test hitch traction by changing the number of medium-weight steel tubes in the drum. At the beginning of the experiment, the soil was plowed by a soil tillage device and leveled by a scraper, and then the test was carried out on a single-wheel test bench. The load on each wheel corresponded to 5 groups of different loads, and each group of tests was repeated twice.

## 3. Results and Discussion

**Figure 4:**
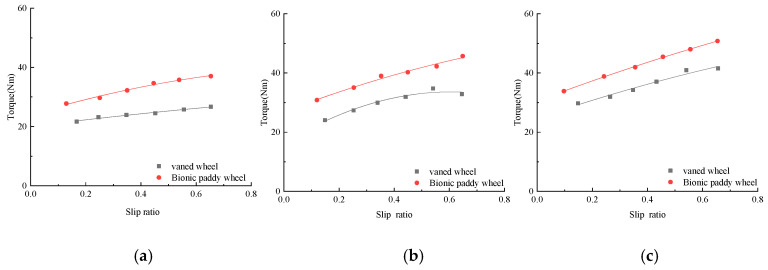
The comparison of torque and slip ratio of different wheels. (**a**) Wheel load 58.57 N, (**b**) wheel load 84.57 N, (**c**) wheel load 107.4 N.

**Figure 5:**
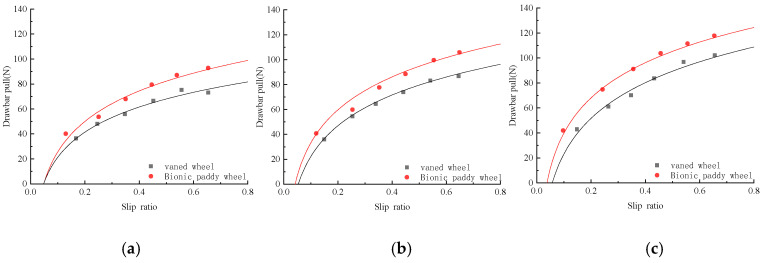
The comparison of drawbar pull and slip ratio of different wheels. (**a**) Wheel load 58.57 N, (**b**) wheel load 84.57 N, (**c**) wheel load 107.4 N.

Torque and hitch tractions are the main factors that affect the vehicle’s traction and pass-ability, and understanding the relationship between them and the slip ratio is helpful for the study of the vehicle’s passing performance. The bionic paddy wheel and the conventional vaned wheel of the tiller were tested in the field according to the test method, and the torque and drawbar pull data obtained in the test were compared, as shown in Figure 4 and Figure 5.

### 3.1. Relationship between Torque and Slip Ratio

Figure 4 is a graph showing the relationship between the torque and the slip ratio of the bionic paddy wheel and the conventional vaned wheel under different wheel loads. It can be seen from Figure 4 that the torque provided by the bionic paddy wheel is larger than that of the conventional vaned wheel under different loads on the wheel, and the larger torque can provide higher starting power. It can be seen from Figure 4 that under different loads on the wheel, the vaned wheel and the bionic paddy wheel have a similar trend; that is, the torque increases with the increase of the slip ratio. When the load on the wheel is 84.57 N, the torque of the conventional vaned wheel begins to decrease after the slip ratio is greater than 0.6, which may be caused by experimental factors. When the loads on the wheel are 58.57 N, 84.57 N and 107.4 N, the maximum torque of the bionic paddy wheel is increased by 38.63%, 31.39% and 22.3% compared with the conventional vaned wheel. The maximum torque of the bionic paddy wheel when the wheel load is 107.4 N is 50.8 N, which is 37.19% and 11.16% higher than that when the wheel load is 58.57 N and 84.57 N, respectively.

### 3.2. Hook Traction and Slip Ratio

Figure 5 is the curve of the relationship between the drawbar pull and the slip ratio of the bionic paddy wheel and the conventional vaned wheel under different wheel loads. It can be seen from Figure 5 that under different loads on the wheel, the drawbar pull of the vaned wheel and the bionic paddy wheel increases first and then tends to be flat with the increase of the slip ratio. When the loads on the wheel are 58.57 N, 84.57 N and 107.4 N, the maximum drawbar pull of the bionic paddy wheel is 92.79 N, 105.85 N and 117.845 N, which are 23.35%, 21.97% and 22.3% higher than that of the conventional vaned wheel. When the on-wheel load of the bionic paddy wheel is 107.4 N, the maximum drawbar pull is increased by 26.99% and 11.33% when the on-wheel load is 58.57 N and 84.57 N. It can be found from Figure 5 that under the same load on the wheel, the change curve of the drawbar pull of the bionic paddy wheel with the slip ratio is always above the conventional vaned wheel, which shows that compared with the conventional vaned wheel, lower bionic paddy wheels promote greater hitch traction. When the slip ratio is the same, the drawbar pull of the bionic paddy wheel can be increased by 20% compared with the conventional vaned wheel, which means that the bionic paddy wheel has better passing performance under the working conditions of high water content in paddy fields.

The curve in Figure 5 is the fitting equation of the curve of drawbar pull of the bionic paddy wheel and the conventional vaned wheel with the slip ratio, which is fitted according to y=a×ln(x)+b. Where x represents the slip ratio, and y represents the drawbar pull of the wheel. Through Figure 5 we can see that as the wheel load increases, the fitting curve also increases, so the coefficients a and b are changed to a function related to the wheel load, and you can get the relationship between the traction and slip ratio of the two-wheel hooks under different wheel loads. where $x_{1}$ represents the wheel load, $x_{2}$ the slip ratio, and y the drawbar pull.

Fitting model of conventional vaned wheel is as Formula (2)

(2) $$y=\left(0.252\times x_{1}+14.24\right)\times ln\left(x_{2}\right)+0.6171\times x_{1}+51.987$$

Bionic walking wheel fitting model as Formula (3)

(3) $$y=\left(0.1128\times x_{1}+28.597\right)\times ln\left(x_{1}\right)+0.5584\times x_{1}+74.219$$

### 3.3. Conclusions

(1) Under the conditions of different loads on the wheel, the maximum torque and hook traction of the bionic paddy wheel and the conventional vaned wheel can increase with the increase of the load on the wheel. The torque of the bionic paddy wheel increases with the increase of the slip ratio under the same round of upper load, and drawbar pull increases gradually with the increase of the slip ratio and then tends to level off. The torque of the conventional vaned wheel increases with the increase of the slip ratio, and drawbar pull increases gradually with the increase of the slip ratio and then tends to level off. These two wheels have similar patterns of change.

(2) The test results comparing the bionic paddy wheel and the conventional vaned wheel show that the torque and hook traction of the bionic paddy wheel are larger than those of the conventional vaned wheel at the same slip ratio. Under the same load on the wheel, the bionic paddy wheel has at least 22% higher torque and 22% higher traction than the conventional vaned wheel, indicating that the bionic paddy wheel can provide higher driving force than the conventional vaned wheel. With hook traction, it can improve the working efficiency of the vehicle in paddy fields.

## Figures and Tables

**Table 1:** Bionic curve fitting parameters.

Coefficient	Bionic Curve A	Bionic Curve B
a	−9.9 × 10^−7^	2.85 × 10^−7^
b	−1.45 × 10^−7^	1.25 × 10^−10^
c	−1.73 × 10^−3^	4.33 × 10^−3^
d	2.76 × 10^−4^	−5.11 × 10^−7^
e	16.69	−18.72
R-square	0.998	0.999
RMSE	0.174	2.51 × 10^−3^

**Table 2:** Test parameters of field soil.

Deformation Index n	Cohesive Defor-mation Modulus of Soil $k_{c}$ $\left(\mathrm{kN}/m^{\left(n+1\right)}\;\right)$	Soil Friction Defor-mation Modulus $k_{\varphi }$ $\left(\mathrm{kN}/m^{\left(n+2\right)}\;\right)$	Cohesion c (Pa)	Internal Friction Angle φ (deg)
0.61	5.27	90.24	2346.2	23.52
